# Supplementary material for: Dualistic Effects of PRKAR1A as a Potential Anticancer Target in Cancer Cells and Cancer-Derived Stem Cells
Source: Int J Mol Sci. 2024 Mar 1;25(5):2876. doi: 10.3390/ijms25052876 (PMC10932193; doi:10.3390/ijms25052876)
Supplement: Supplementary file 1 [file ijms-25-02876-s001.zip › ijms-2859422-supplementary.pdf]

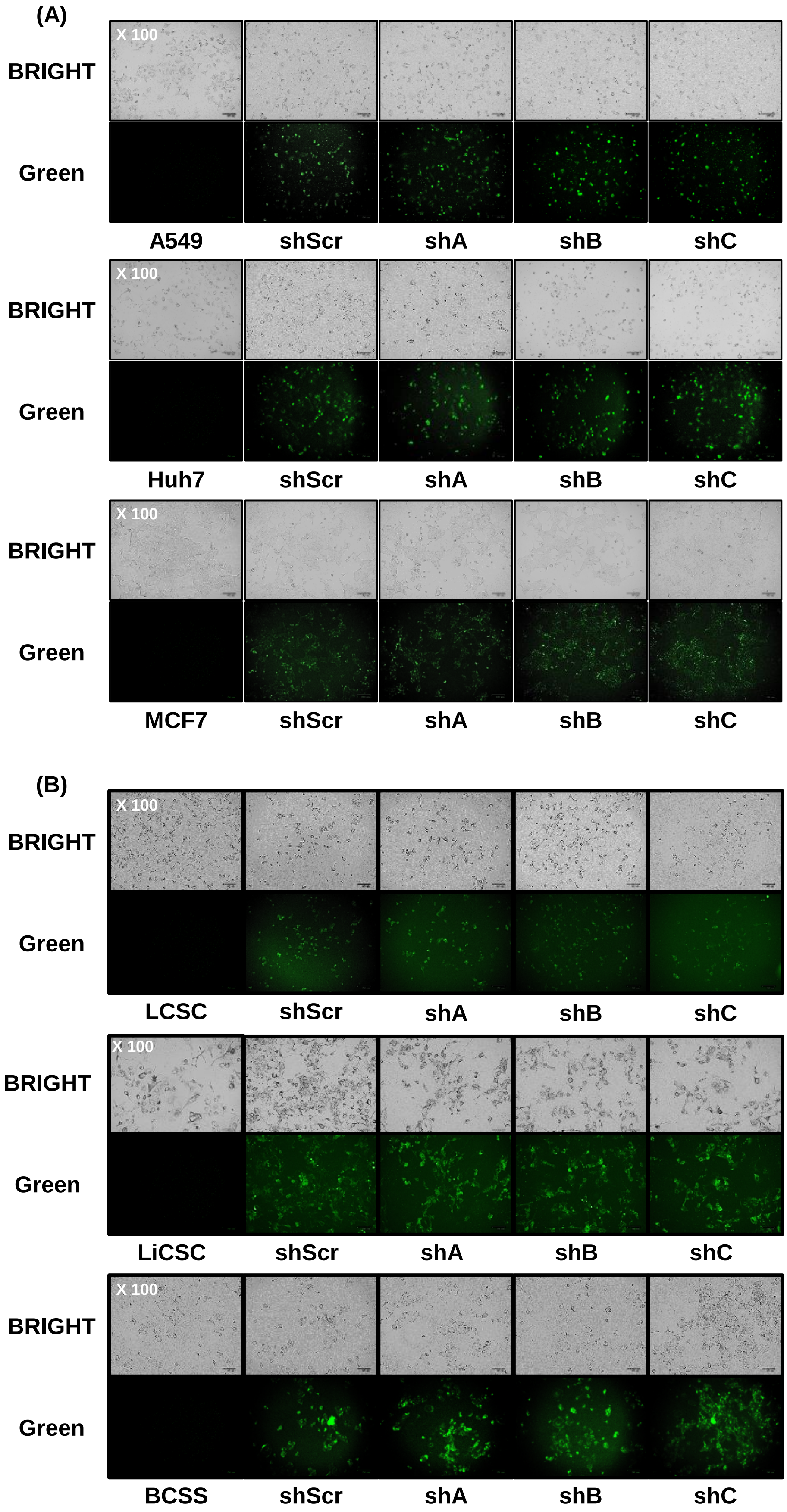

Figure S1. Transfection efficiency and confirmation of shRNA series by GFP expression in cancer cells and cancer stem cells. Three types of cancer cells (A) and Each cancer cells-derived cancer stem cells (B)

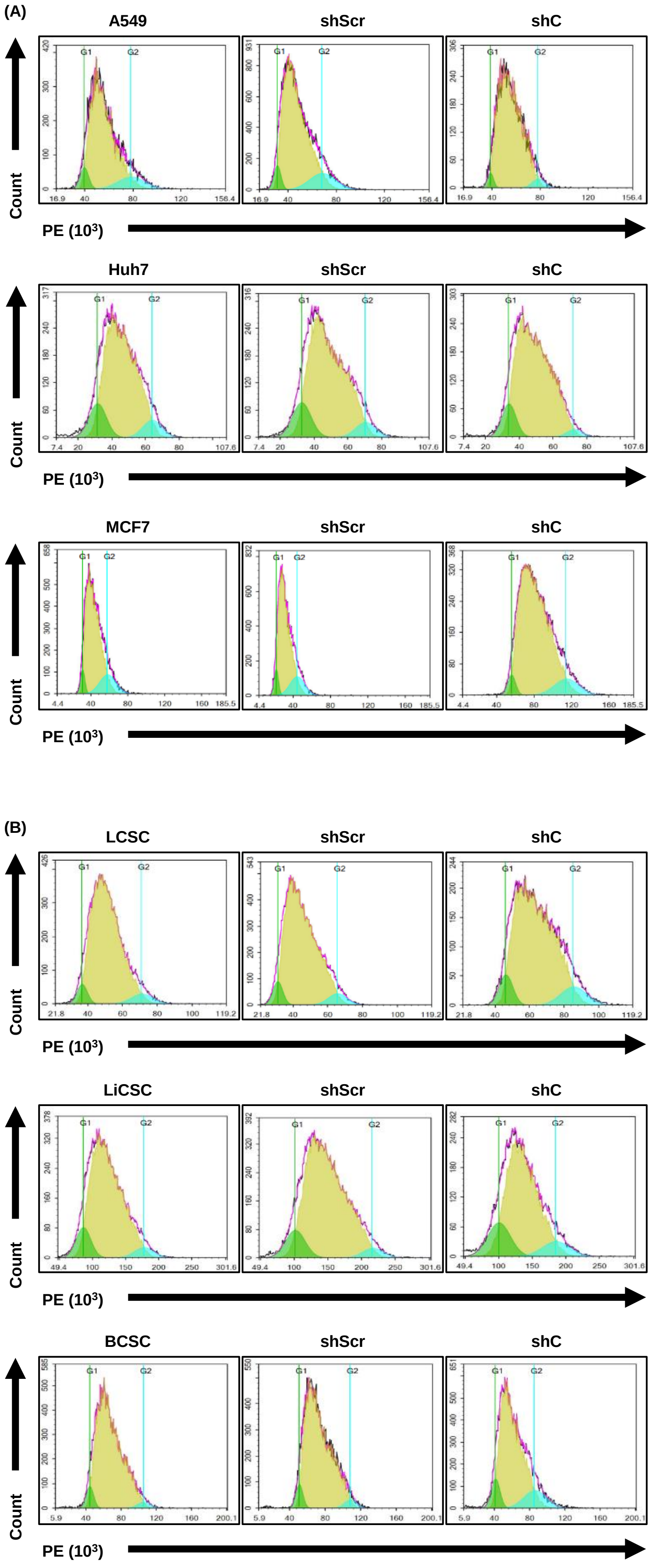

Figure S2. Cell cycle analysis by flow cytometry post-PRKAR1A shRNA treatment. Three types of cancer cells (A) and Each cancer cells-derived cancer stem cells (B)

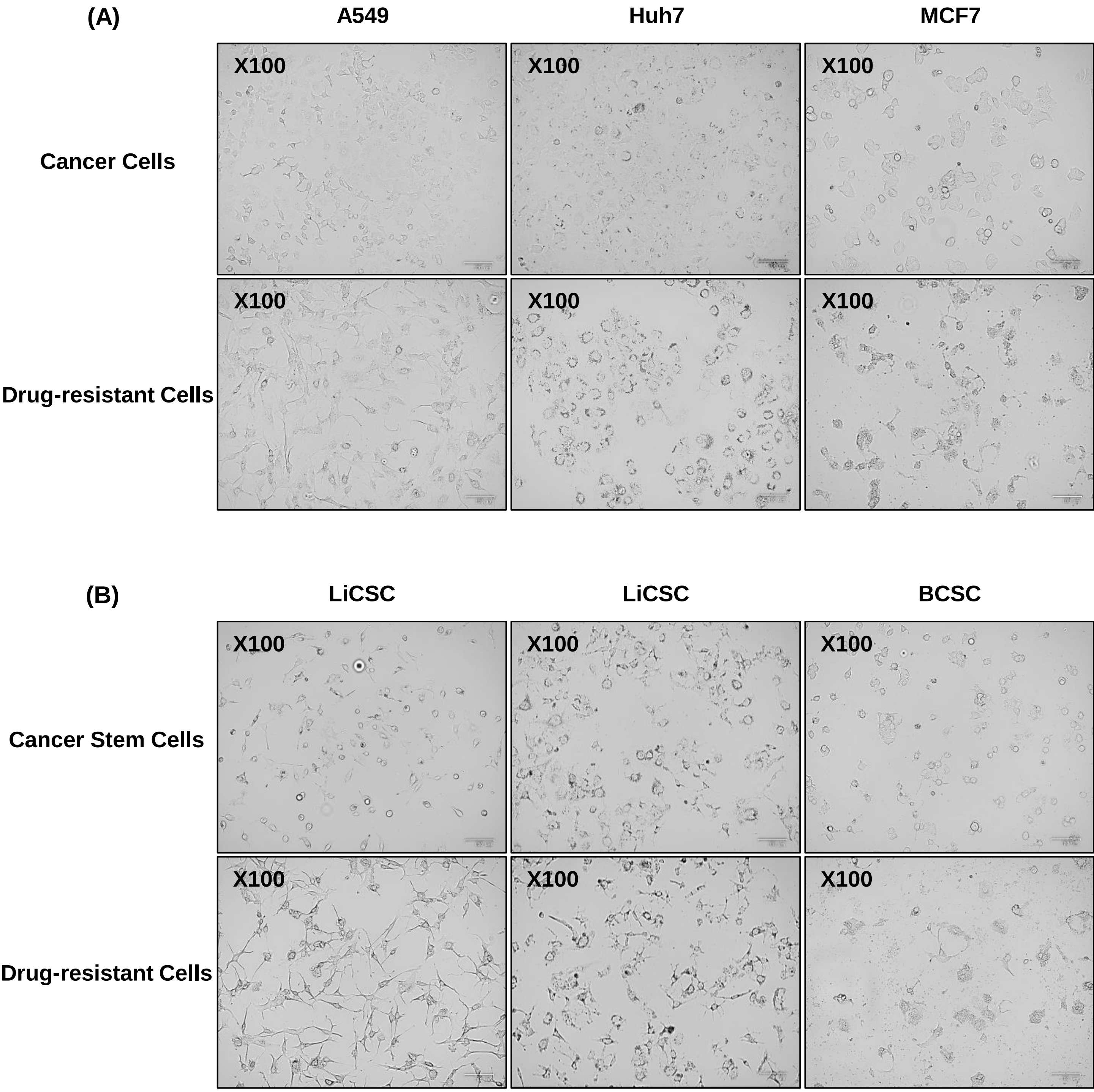

Figure S3. Bright image of generated drug-resistant cells.

Non-drug resistant and drug resistant cancer cells generated against anticancer agent (A) and Non-drug resistant and drug resistant cancer stem cells generated against anticancer agent (B)
